# The Prognostic Impact of Histology in Esophageal and Esophago-Gastric Junction Adenocarcinoma

**DOI:** 10.3390/cancers13205211

**Published:** 2021-10-18

**Authors:** Roberto Fiocca, Luca Mastracci, Marialuisa Lugaresi, Federica Grillo, Antonietta D’Errico, Deborah Malvi, Paola Spaggiari, Anna Tomezzoli, Luca Albarello, Ari Ristimäki, Luca Bottiglieri, Elena Bonora, Kausilia K. Krishnadath, Gian Domenico Raulli, Riccardo Rosati, Uberto Fumagalli Romario, Giovanni De Manzoni, Jari Räsänen, Sandro Mattioli

**Affiliations:** 1 Department of Surgical and Diagnostic Sciences (DISC), University of Genova, 16125 Genova, Italy; luca.mastracci@unige.it (L.M.); federica.grillo@unige.it (F.G.); 2 Unit of Anatomic Pathology, Ospedale Policlinico San Martino IRCCS, 16125 Genova, Italy; 3 Department of Medical and Surgical Sciences (DIMEC), Alma Mater Studiorum, University of Bologna, 40126 Bologna, Italy; marialuisa.lugaresi2@unibo.it (M.L.); elena.bonora6@unibo.it (E.B.); sandro.mattioli@unibo.it (S.M.); 4 Division of Thoracic Surgery, Maria Cecilia Hospital, GVM Care & Research Group, Cotignola, 48022 Ravenna, Italy; 5 Pathology Unit, IRCCS Azienda Ospedaliero-Universitaria di Bologna, 40126 Bologna, Italy; antonietta.derrico@unibo.it (A.D.); deborah.malvi@aosp.bo.it (D.M.); 6 Department of Experimental, Diagnostic and Specialty Medicine (DIMES), Alma Mater Studiorum, University of Bologna, 40126 Bologna, Italy; 7 Unit of Anatomic Pathology, Humanitas University, 20089 Milan, Italy; paola.spaggiari@humanitas.it; 8 Unit of Anatomic Pathology, Azienda Ospedaliera di Verona, 37122 Verona, Italy; anna.tomezzoli@aovr.veneto.it; 9 Pathology Unit, San Raffaele Scientific Institute, 20135 Milan, Italy; albarello.luca@hsr.it; 10 Department of Pathology, HUSLAB and HUS Diagnostic Center, University of Helsinki, 00170 Helsinki, Finland; ari.ristimaki@hus.fi; 11 Helsinki University Hospital, 00170 Helsinki, Finland; 12 Unit of Anatomic Pathology, Istituto Europeo di Oncologia, 20122 Milan, Italy; luca.bottiglieri@ieo.it; 13 Unit of Medical Genetics, IRCCS Azienda Ospedaliero-Universitaria di Bologna, 40126 Bologna, Italy; 14 Laboratory of Experimental Medicine and Pediatrics (LEMP), Department of Gastroenterology and Hepatology, University Hospital Antwerp, 2650 Antwerp, Belgium; Sheila.Krishnadath@uza.be; 15 Pathology Unit, AUSL Area Vasta Romagna, 48100 Ravenna, Italy; g.raulli@ausl.ra.it; 16 Department of Gastrointestinal Surgery, San Raffaele Hospital, Vita-Salute San Raffaele University, 20135 Milan, Italy; rosati.riccardo@hsr.it; 17 Digestive Surgery, European Institute of Oncology, IRCCS, 20122 Milan, Italy; uberto.fumagalliromario@ieo.it; 18 Department of Surgery, General and Upper G.I. Surgery Division, University of Verona, 37126 Verona, Italy; giovanni.demanzoni@univr.it; 19 Department of General Thoracic and Esophageal Surgery, Helsinki University Hospital, 00170 Helsinki, Finland; jari.rasanen@hus.fi

**Keywords:** esophageal adenocarcinoma, esophago-gastric junction adenocarcinoma, histology, prognosis

## Abstract

**Simple Summary:**

Esophageal and esophago-gastric junction adenocarcinomas (EA/EGJAs) are a heterogeneous group of cancers. Stage is the most important prognostic factor, while morphology, determined by histologic analysis, has up until now played a minor role. Even new molecular classifications (which should be based on accurate histologic assessment) are a long way off from being used in day to day practice. The reassessment of nearly 300 EA/EGJAs enabled us to re-evaluate morphology and identify a two-tiered grading approach in glandular adenocarcinomas (80%) based on a cut off of 6% of poorly differentiated components (well differentiated versus poorly differentiated). Furthermore, rare, but prognostically significant, variants were recognized with an in-depth morphologic description. On this basis, two morphologic risk groups (lower risk and higher risk) were identified, adding significant prognostic value to the stage. The accurate morphologic description of EA/EGJAs must be a prerequisite for a better understanding of prognosis, molecular events, and response to treatment.

**Abstract:**

Stage significantly affects survival of esophageal and esophago-gastric junction adenocarcinomas (EA/EGJAs), however, limited evidence for the prognostic role of histologic subtypes is available. The aim of the study was to describe a morphologic approach to EA/EGJAs and assess its discriminating prognostic power. Histologic slides from 299 neoadjuvant treatment-naïve EA/EGJAs, resected in five European Centers, were retrospectively reviewed. Morphologic features were re-assessed and correlated with survival. In glandular adenocarcinomas (240/299 cases—80%), WHO grade and tumors with a poorly differentiated component ≥6% were the most discriminant factors for survival (both *p* < 0.0001), distinguishing glandular well-differentiated from poorly differentiated adenocarcinomas. Two prognostically different histologic groups were identified: the *lower risk group,* comprising glandular well-differentiated (34.4%) and rare variants, such as mucinous muconodular carcinoma (2.7%) and diffuse desmoplastic carcinoma (1.7%), versus the *higher risk group,* comprising the glandular poorly differentiated subtype (45.8%), including invasive mucinous carcinoma (5.7%), diffuse anaplastic carcinoma (3%), mixed carcinoma (6.7%) (CSS *p* < 0.0001, DFS *p* = 0.001). Stage (*p* < 0.0001), histologic groups (*p* = 0.001), age >72 years (*p* = 0.008), and vascular invasion (*p* = 0.015) were prognostically significant in the multivariate analysis. The combined evaluation of stage/histologic group identified 5-year cancer-specific survival ranging from 87.6% (stage II, lower risk) to 14% (stage IVA, higher risk). Detailed characterization of histologic subtypes contributes to EA/EGJA prognostic prediction.

## 1. Introduction

Esophageal cancer is the sixth highest cause of cancer related death and the eighth most common cancer worldwide [1,2,3]. A shift in epidemiology is taking place in high-income western countries with adenocarcinoma incidence surpassing squamous cell carcinoma [4,5,6,7]. Five-year overall survival for esophageal adenocarcinoma (EA)/esophago-gastric junction adenocarcinoma (EGJA) is around 20%; 5-year survival rates are less than 45%, even for patients in the curative stage and those treated with a multi-modal approach [2,3,8].

One of the possible reasons for the relative ineffectiveness of EA/EGJA treatment is that guidelines for diagnosis, surgical classification, staging, and therapy are based on clinical research, indistinctly performed on a biologically heterogeneous group of neoplasms, uniformly defined as “adenocarcinoma” [9,10]. Furthermore, clinical staging, even though performed using advanced radiologic and endoscopic techniques, over- or under-estimates 40% of cases [11,12].

In short, the most powerful prognostic parameters driving therapy are, to date, clinical and pathological TNM staging—the former is far from reliable, while the latter is derived from the surgical specimen, detailed after resection, when the core of therapy (i.e., surgery or neoadjuvant therapy + surgery) is already over [13,14,15,16,17,18]. To overcome these limits, other prognostic features have been explored, including clinic–pathologic associations with Barrett’s or gastric intestinal metaplasia [19,20,21,22] and molecular subtyping [23,24]. These studies have added important pieces to the puzzle, however, as yet, they have not found practical application in patient management.

With regards to pathologic factors, the most important feature in EA/EGJAs (other than stage), is grade, identified in the current WHO Classification of Tumors 2019 [25] as a three-tiered system, defined by the percentage of gland-forming areas. This three-tiered approach influences EA/EGJA prognostic grouping, at least in early lesions without nodal metastases [26]. It is worth noting that both gastric and colorectal cancers have shifted to a more reproducible, two-tiered, low/high grade, system [27,28,29].

EA/EGJAs are described (according to the WHO Classification of Tumors 2019) as having tubular or papillary architecture, while mucinous and signet ring cell (SRC) patterns are rarely present as pure forms. Limited evidence for the prognostic relevance of histologic subtypes is available for EA/EGJAs [25]; therefore, patterns should be described but no specific definition of histotypes is required. Rare, prognostically relevant histotypes, seen in gastric cancer, such as muconodular adenocarcinoma [30], have never been comprehensively described/studied in EA/EGJAs.

Laurén’s classification, though originally proposed for gastric cancer [31], has also been applied to EAs and several reports have outlined that Laurén’s diffuse type and SRC component are associated with more aggressive behavior and worse survival compared to Laurén’s intestinal type, thus suggesting that therapeutic strategies should be tailored according to these features [32,33].

The purpose of this study was to re-assess a large case series of EA/EGJAs primarily submitted to surgery in order to: (1) identify histologic prognostic features; (2) evaluate the prognostic role of grading in advanced, surgically resected glandular subtypes; (3) identify other morphologic subtypes, especially with regards to their frequency and prognosis.

## 2. Materials and Methods

### 2.1. Study Population

Patients with EA/EGJAs, submitted to radical surgical resection (between 2003–2019), were retrospectively recruited from the databases of five institutions belonging to the Esophageal Adenocarcinoma Study Group Europe (EACSGE).

Inclusion criteria were: age >18 years; histological diagnosis of adenocarcinoma; availability of endoscopy/barium swallow reports for Siewert’s classification; availability of the original pathology report, stained slides, and/or paraffin blocks from the surgical specimen; post-surgical follow-up data, including overall (OS) and cancer-specific (CSS) and disease-free (DFS) survival.

Exclusion criteria were pT1a/N0/M0 cases according to the AJCC 8th edition [26] and the following rare histotypes: adenosquamous, neuroendocrine carcinomas, or mixed neuroendocrine–non-neuroendocrine carcinomas (demonstrated by immunohistochemical expression of neuroendocrine markers). No patient treated with neoadjuvant therapy was included to ensure that tumor morphology was free from treatment-induced factors (e.g., fibrosis, loss of architectural features, post-treatment mucinous areas).

Indications for primary surgery followed international guidelines valid during the study period [34,35], except for a group of cases cT2−4/N+ (peritumoral) resected before 2015 by one of the surgical groups, according to approved study protocols [21,36]. After surgery, systemic treatment followed current guidelines in relation to stage or, in the case of recurrence, according to the patient’s will [34,35,37].

### 2.2. Data Collection

Clinical data of patients were transferred from surgical groups’ databases into the study files in anonymous form, and maintained for all subsequent transfers/analyses. Data on gender, age, clinical stage, Siewert’s classification, pathological stage/pTNM [26], lymph node number, metastatic lymph node number, lymph node ratio, margin status, adjuvant therapy, follow up (months), cancer relapse, and cause of death were collected.

### 2.3. Histologic Variables

In a first meeting, four expert gastrointestinal pathologists (RF, DM, ADE, LM) evaluated a set of 80 cases and defined the histologic characteristics of interest: WHO architectural patterns [25], Laurén’s type (intestinal versus diffuse versus unclassified morphology), Ming’s expanding versus infiltrative growth [38], percentage of loss of glandular structure with maintained intercellular cohesion (including poorly differentiated clusters), grade according to WHO Classification of Tumors 2019 (G1 well-formed glands in >95%, G2 in 50–95%, G3 in <50% of tumor), percentage of cohesive versus non-cohesive neoplastic cell component, percentage of SRCs [39] (defined as cells with ample cytoplasmic mucin, optically clear on hematoxylin and eosin, and an eccentrically placed nucleus), percentage of extracellular mucinous component, and presence of perineural and vascular invasion.

During a second meeting, all involved pathologists reviewed selected cases, with the aim of sharing and standardizing the aforementioned histologic characteristics. Pathologists were required to pre-select 1 to 3 representative hematoxylin and eosin-stained slides of both the superficial and the invasive component of EA/EGJAs. For each case, 1–2 corresponding blocks of formalin-fixed paraffin-embedded tissue were also made available. All remaining collected, not previously analyzed, cases were centrally reviewed (RF, LM).

### 2.4. Definition of Rare Histotypes

Within the entire cohort, consisting mostly of adenocarcinomas with glandular features (tubular/papillary/solid patterns), tumors with mucinous, signet ring/non-cohesive cells and mixed features were identified. As little information is available in the literature for rare EA/EGJA histotypes, similarities with rare gastric cancer histotypes were searched. In particular, with regards to mucinous neoplasms, a mucinous muconodular carcinoma (MMC) subtype [30], defined as tumors showing an “over-whelming (80% or more) mucinous component in the form of well-demarcated mucin lakes with tumor cells floating inside, predominance of extracellular mucin over tumor cells, an overall expanding pattern of growth, with moderate cellular anaplasia”, was identified. Such MMCs were separated from the remaining invasive mucinous carcinomas (IMC), which showed an infiltrative growth pattern [40], higher cellularity, and more pronounced cellular atypia. As previously described, in both these tumor types, SRC may be found floating in lakes of extracellular mucin, but these aspects should not lead to a diagnosis of SRC carcinoma [39,41].

Non-cohesive cell carcinomas, were separated into diffuse desmoplastic carcinomas (DDC), defined as “non cohesive carcinomas with fibroblast-rich desmoplasia surrounding individual (or minute groups of), moderately atypical, tumor cells with limited cellularity”, and diffuse anaplastic carcinomas (DAC) characterized by “poorly cohesive, large- to medium-sized cells with large, pleomorphic, highly atypical nuclei, prominent nucleoli, high proliferative rates, high cellularity and scarce stroma” [42]. SRC have been described in both cancer types, and these are more abundant in the superficial portion of DDC [42], while they are variably found in DAC.

Mixed adenocarcinomas (MIX) were defined as tumors displaying two or more distinct histologic components (glandular/tubular/papillary and poorly cohesive/signet ring) according to gastric adenocarcinoma WHO criteria (as none are specifically available for EA/EGJA cancers) [41].

### 2.5. Statistical Analysis

Data were represented as the median and interquartile range (IQR) for continuous variables and as a number (%) for categorical variables. The χ2 test or Fisher’s test (expected number less than 5) and the Mann–Whitney test were used to analyze categorical and continuous variables, respectively. The χ2 test for given probabilities [R software, version 3.4.3 (30 November 2017) Project for Statistical Computing, Vienna, Austria], a particular test useful in the presence of small samples, was adopted applying the Bonferroni’s correction. The most prognostically relevant cut off of the poorly differentiated component in glandular adenocarcinoma was preliminarily defined by ROC analysis.

Survival analyses (OS, CSS, DFS) were performed using the Kaplan–Meier method and the log-rank test. Univariate and multivariate (forward stepwise conditional method) Cox regression analyses were performed to estimate the effects of pathological parameters on CSS. Only the variables detected to be statistically significant in the univariate analysis were entered in the multivariate analysis. In the stepwise procedure, significance levels of 0.05 for entering and 0.10 for removing the respective explanatory variables were used to determine the independent risk factors.

A *p*-value < 0.05 was considered significant. Statistical analyses were performed using the SPSS15.0 software package (SPSS Inc., Chicago, IL, USA) and R [version 3.4.3 (30 November 2017) Project for Statistical Computing, Vienna, Austria].

The study was conducted in accordance with the ethical standards of the Helsinki Declaration (1964, amended in 2008) and of the World Medical Association. The institutional ethics review board for IRST IRCCS Area Vasta Romagna CEIIAV- Italy, approved this study (# L3P1223) (Reg. Sper. 109/2016 Protocol 7353/51/2016). Consent was obtained when possible.

## 3. Results

Three hundred and forty-two surgical specimens of EA/EGJAs were accrued. Early pT1/N0/M0 adenocarcinomas (43 cases) were excluded, while five pT1 cases with lymph node metastases were retained.

Two hundred and ninety-nine cases (243 males/56 females, median age 72 years—IQR 62–78) were evaluated. A summary of the clinical and pathologic features is shown in Table 1. Except for eight p-stage IB cases (all T2-N0), the others were stage II (72 cases—24.1%), stage III (149 cases—49.8%), or stage IVA (70 cases—23.4%).

The prevalent cancer growth pattern according to Ming was infiltrative with only 28.1% (84) of cases growing with an expanding border. The majority of adenocarcinomas were classified according to Laurén’s classification as intestinal type (250 cases—83.6%), and a minority were classified as diffuse type (29 cases—9.7%) or were unclassified (20 cases—6.7%). Signet ring cell components (ranging from a minimum of 5% to a maximum of 90% of neoplastic cells) were identified in 53 cases (17.7%), 25 of which showed >15% SRCs.

### 3.1. Grade and Percentage of Loss of Glandular Structure

The analysis of grade, according to WHO Classification of Tumors 2019 criteria, was applied exclusively to adenocarcinomas with tubular/papillary architecture (240/299 cases —80.2%). Grade 1 adenocarcinomas accounted for 43% (103/240), grade 2 for 27.5% (66/240), and grade 3 for 29.5% (71/240).

**Figure 1:**
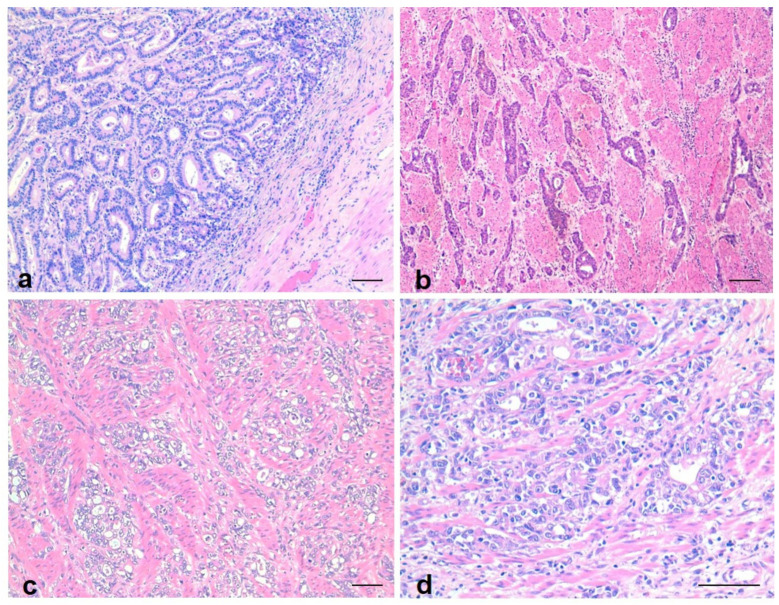
The glandular well-differentiated (GL-WD) subtype is characterized by well-formed glands and can present either an expanding (**a**) or infiltrative (**b**) growth pattern; loss of glandular structure (i.e., poorly differentiated component) may be seen in <6% of the tumor area. (**c**,**d**) Glandular poorly differentiated (GL-PD) subtype: two examples of the poorly differentiated component showing loss of glandular structure with preserved cell cohesion; adjacent to these areas, more glandular structures can be recognized in variable percentage. This pattern characterizes GL-PD, it shows an infiltrative growth pattern and is usually found in the deep portion of the tumor (hematoxylin and eosin; scale bars 100 microns).

ROC analysis identified a cut off of 6% of loss of glandular structure (i.e., poorly differentiated component) to be the most informative with regards to survival. On this basis, 103 cases with <6% of poorly differentiated components were considered low grade (which corresponded to all G1 cases; Figure 1a,b), while 137 cases with ≥6% of poorly differentiated components were considered high grade (which corresponded to G2 and G3 cases; Figure 1c,d).

### 3.2. Frequency of Rare Histotypes

**Figure 2:**
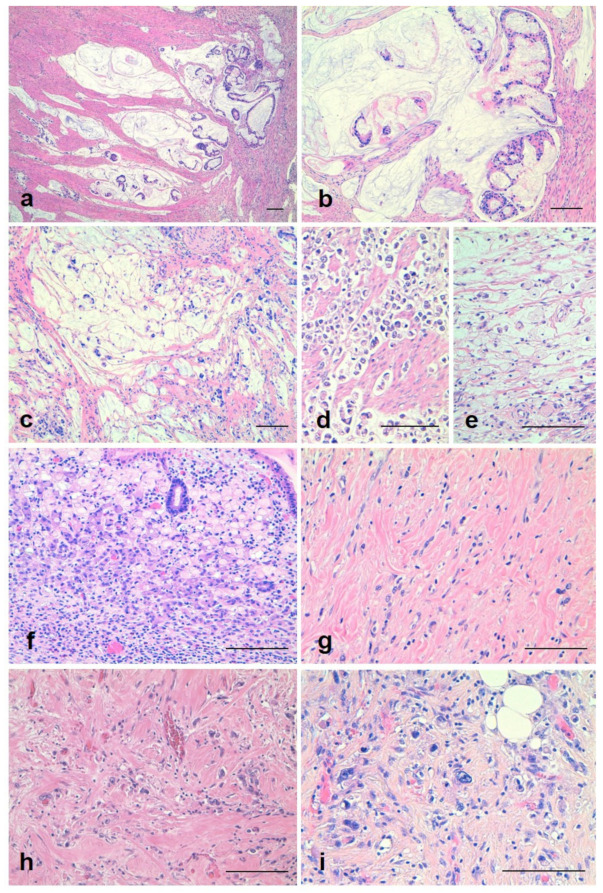
Rare EA/EGJA histotypes. (**a**,**b**) Mucinous muconodular carcinoma (MMC) subtype: the mucinous component is characterized by glands or strips of cohesive cells floating within extracellular mucin lakes; the growth pattern is exclusively expanding. (**c**–**e**) Infiltrative mucinous carcinoma (IMC) subtype: poorly cohesive tumor cells, isolated or in small aggregates, show an infiltrative pattern (**c**) with a variable degree of atypia (**d**) and may have signet ring cell features (**e**). (**f**,**g**) Diffuse desmoplastic carcinoma (DDC) subtype: poorly cohesive cells, isolated or in small aggregates, infiltrate the gastric wall, producing marked desmoplasia. Signet ring cells are often limited to the most superficial part of the tumor (**f**), while the deep part shows infiltrative growth, mild cytological atypia, and few mitoses (**g**). (**h**,**i**) Diffuse anaplastic carcinoma (DAC) subtype: tumor growth pattern is infiltrative with poorly cohesive cells (**h**), but, unlike the “diffuse desmoplastic type”, cells show a high degree of cytological atypia (**i**) and numerous mitoses. Signet ring cells, which can be variably seen, are not a major feature of this subtype (hematoxylin and eosin; scale bars 100 microns).

**Figure 3:**
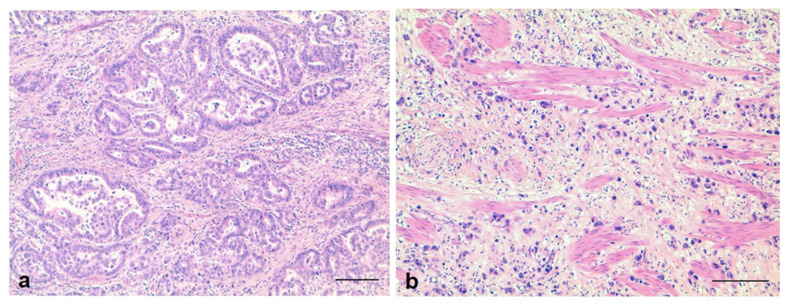
Mixed subtype shows an admixture of frankly glandular aspects (**a**) and a poorly cohesive cellular component (**b**). Figure (**a**,**b**) belong to different areas of the same tumor and show the simultaneous presence of a differentiated glandular and a poorly cohesive component (hematoxylin and eosin; scale bars 50 microns).

Fifty-nine cases (19.7%) were classified as rare morphologic subtypes, including: 8 mucinous muconodular carcinomas—MMC (Figure 2a,b); 17 invasive mucinous carcinomas—IMC (Figure 2c–e); 5 diffuse desmoplastic carcinomas—DDC (Figure 2f,g); 9 diffuse anaplastic carcinomas—DAC (Figure 2h,i); 20 mixed carcinomas (Figure 3a,b).

### 3.3. Survival Analysis

The median follow-up time was 25 months (IQR 16–51). Comprehensive analysis showed that the 5-year OS was 40.8%, CSS was 47.8%, and DFS was 43%. As expected, cancer stage significantly impacted on OS, CSS, and DFS with *p* < 0.0001 (Table 2).

**Figure 4:**
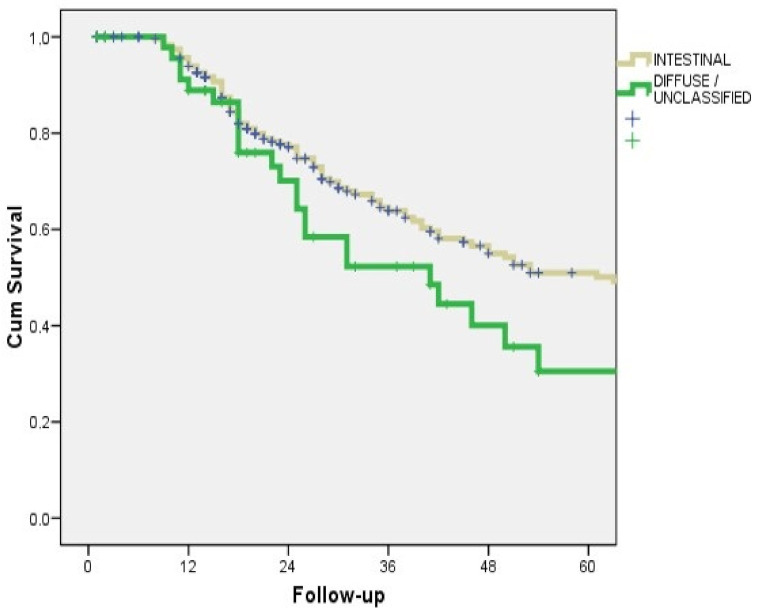
Kaplan–Meier CSS curve for patients with intestinal and diffuse/unclassified histology according to the Laurén’s classification: 5-year CSS was 51% for intestinal subtype (250 cases) and 30.5% for diffuse/unclassified subtype (49 cases) (*p* = 0.089).

Survival analysis in relation to Laurén’s classification did not show significant differences (Figure 4), neither considering intestinal versus diffuse versus unclassified types separately nor grouping diffuse/unclassified versus intestinal types. The WHO Classification of Tumors 2019 classification [25], including tubular, papillary, mucinous, poorly cohesive/signet ring cell, and mixed histotypes, showed no correlation with survival (*p* = 0.325).

### 3.4. Survival Analysis of Glandular Adenocarcinomas

**Figure 5:**
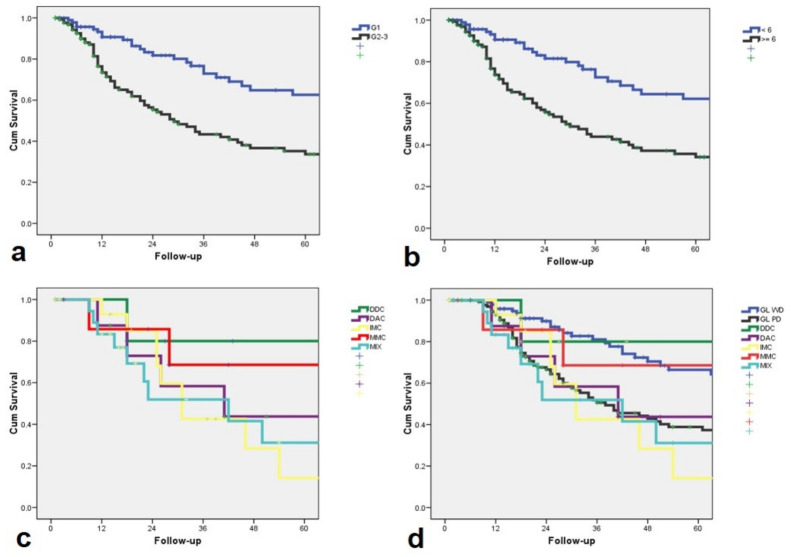
(**a**) Kaplan–Meier cancer-specific survival (CSS) curve according to the WHO grading system for patients with G1 (103 cases) versus G2–G3 (137 cases). The difference in survival was highly significant (*p* < 0.0001). (**b**) CSS curve for patients with glandular adenocarcinoma divided according to 6% cut-off of poorly differentiated components identified by ROC (<6% 105 cases—GL-WD, ≥6% 135 cases—GL-PD). The difference in survival was highly significant (*p* < 0.0001). (**c**) CSS curves of rare histotypes only and (**d**) CSS curves of all histotypes, including glandular well-differentiated and glandular poorly differentiated subtypes. Abbreviations: GL-WD—glandular well-differentiated subtype; GL-PD—glandular poorly differentiated subtype; MMC—mucinous muconodular carcinoma; IMC—invasive mucinous carcinoma; DDC—diffuse desmoplastic carcinoma; DAC—diffuse anaplastic carcinoma; MIX—mixed carcinoma.

Regarding only glandular adenocarcinomas, the percentage of the poorly differentiated component (*p* < 0.0001), Ming’s infiltrative growth pattern (*p* = 0.011), vascular invasion (*p* = 0.001), and perineural invasion (*p* < 0.0001) negatively impacted on CSS in the univariate analysis. Grade (G1 versus G2 versus G3) according to WHO Classification of Tumors 2019 was a good predictor of survival (*p* < 0.0001) (Figure 5a). When differences in survival between G2 versus G3 alone were analyzed, this was statistically significant (*p* = 0.013), but with lower statistical inference. The 6% cut off for the poorly differentiated component identified by ROC curve analysis, predicted survival equally as well as grade, but with only two tiers (*p* < 0.0001) (Figure 5b).

Multivariate analysis was carried out considering all the prognostically significant features identified at univariate analysis. At multivariate analysis, ≥6% of poorly differentiated components (used to separate low-grade/well-differentiated (GL-WD) versus high-grade/poorly differentiated (GL-PD) glandular adenocarcinomas) was the feature best correlated with survival (*p* = 0.010), while vascular invasion and perineural invasion also proved to be statistically significant but with lower statistical inference (*p* = 0.036 and *p* = 0.040, respectively) (Table 2).

### 3.5. Survival Analysis of Rare Histotypes

Regarding rare histotypes, survival curves identified two histologic subtypes, namely MMC and DDC, with better prognoses compared to the others. In contrast, the biological behavior of IMC, DAC, and mixed carcinomas was more aggressive and comparable to that of high-grade glandular adenocarcinomas (Figure 5c,d). Although the survival curves of the rare histotypes showed different survival rates, these were not statistically significant, probably due to the small number of such cases. However, using the χ2 test for given probabilities (useful for small samples), and applying the Bonferroni’s correction, a significant difference in the frequency of mortality between MMC and DDC versus IMC, DAC, and mixed carcinomas (*p* = 0.0125) was detected.

### 3.6. Survival and Systemic Treatment

Systemic treatment was started in 116/299 patients (38.7%) because of advanced pTNM stage or because of relapse of disease; treatment was interrupted in 14 patients (12%), while 102 (88%) patients underwent the planned protocol. Kaplan–Meier CSS analysis showed no statistically significant differences between patients who did or did not undergo adjuvant therapy in relation to the pathological subtype or pTNM stage.

### 3.7. Prognostic Histologic Groups

**Figure 6:**
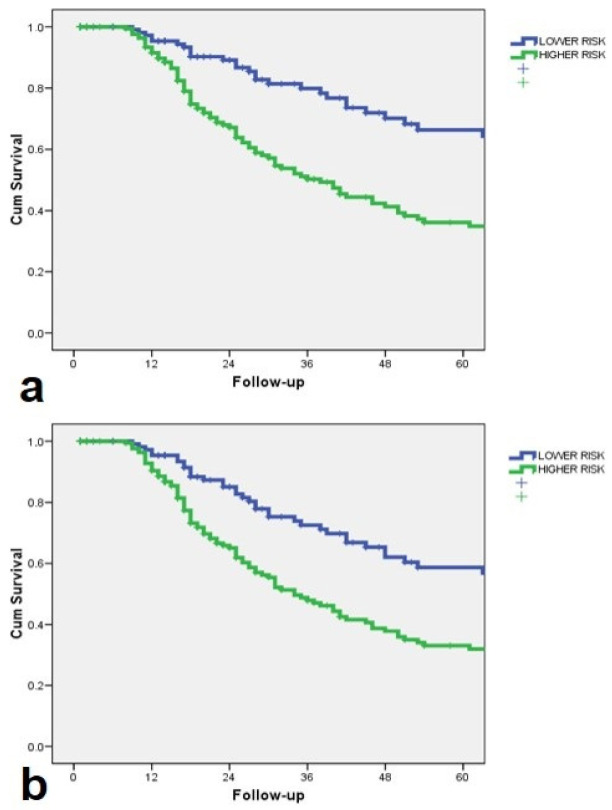
Kaplan–Meier CSS (**a**) and DFS (**b**) curve for patients with lower risk carcinomas (GL-WD, MMC, DDC) (116 cases) and higher risk carcinomas (GL-PD, IMC, DAC, MIX carcinoma) (183 cases) (*p* < 0.0001 and *p* = 0.001 respectively). 5-year CSS was 66.3% for lower risk carcinomas and 36.1% for higher risk carcinomas. Abbreviations: GL-WD—glandular well-differentiated subtype; GL-PD—glandular poorly differentiated subtype; MMC—mucinous muconodular carcinoma; IMC—invasive mucinous carcinoma; DDC—diffuse desmoplastic carcinoma; DAC—diffuse anaplastic carcinoma; MIX—mixed carcinoma.

Kaplan–Meier 5-year survival for patients classified according to the seven histologic subtypes (glandular and rare histotypes together) showed CSS of 66.4% for GL-WD adenocarcinoma (103 cases), 38.9% for GL-PD adenocarcinoma (137 cases), 68.6% for MMC (8 cases), 14.2% for IMC (17 cases), 80% for DDC (5 cases), 43.8% for DAC (9 cases), and 31.2% for mixed carcinoma (20 cases) (*p* = 0.003) (Figure 5d). On the basis of all these findings, two histologic subtype groups with a clearly different prognosis were identified: lower risk carcinomas (including GL-WD, MMC, and DDC subtypes) and higher risk carcinomas (including GL-PD, IMC, DAC, and mixed subtypes) (Figure 6a,b). The survival of these two groups was significantly different in terms of CSS (*p* < 0.0001), DFS (*p* = 0.001), and OS (*p* < 0.0001).

### 3.8. Survival Analysis of the Entire Cohort

Univariate analysis on all the case series showed that age (>72 years), presence of the SRC component in at least 5% of neoplastic cells, presence of any non-cohesive cancer cell component, infiltrative growth pattern according to Ming, and presence of vascular and perineural invasion negatively impacted on CSS (Table 3) (*p* < 0.05).

When matching all the considered parameters against CSS in the multivariate analysis, only four of these maintained a significant prognostic impact: stage (*p* < 0.0001), histologic classification (higher risk versus lower risk; *p* = 0.001), patient age > 72 years (*p* = 0.008), and vascular invasion (*p* = 0.015) (Table 3).

**Scheme 1:**
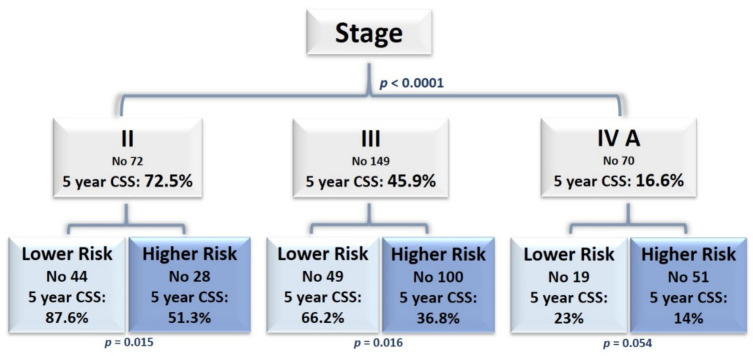
Distribution of patients according to pathologic stage, cancer-specific survival (CSS), and lower/higher risk histologic types.

Finally, the combined evaluation of stage and histological subtypes (lower versus higher risk) showed a significant prognostic discrimination, with values of 5-year CSS ranging from 87.6% in the stage II lower risk group to 14% in the stage IVA higher risk group (Scheme 1).

## 4. Discussion

According to the WHO Classification of Tumors 2019 [25], reappraised in the AJCC 8th edition staging system, grade is a fundamental part of the prognostic assessment of EA/EGJAs, while little emphasis is given to rare histotypes. The present study, on a large series of EA/EGJA cases submitted to surgery (without neoadjuvant treatment), has considered numerous histologic parameters that have already been proven to be of prognostic impact, either in the esophagus and/or in the stomach. Amongst these, grade, vascular and perineural invasion, Ming’s infiltrative growth pattern, and signet ring/non-cohesive cell components have proved to be statistically significant in relation to outcome, at least in univariate analyses. In contrast, Laurén’s classification, which is widely adopted, has not shown prognostic significance, even though a trend towards significance was seen.

While the WHO Classification of Tumors 2019 of gastric adenocarcinoma specifies that grading applies primarily to tubular and papillary adenocarcinoma, and not to other subtypes, no such caveat is present for EA/EGJAs. Our contribution has shown that grade is the principal prognostic factor in EA/EGJAs with glandular architecture (similarly to gastric cancer), while in other rare histotypes, subtyping seems to have a greater impact. Loss of glandular structure in >6% of the tumor area proved to be the most informative indicator for survival, and hence was used to separate advanced adenocarcinomas into well-differentiated (GL-WD) and poorly differentiated (GL-PD) subtypes. This distinction is particularly important as the vast majority of EA/EGJAs belong to the glandular phenotype. Though a three-tiered grading system is advocated in early cancers, as it is used to stratify prognostic groups according to AJCC 8th edition, further study is required to assess whether the two-tiered system performs as well in early EA/EGJAs.

Rare histotypes are less frequent compared to gastric cancer, making up only 13% of our case series in their pure form, while a further 6.7% were morphologically mixed. This implies that extremely large series of EA/EGJAs must be accrued to increase numerosity of these subtypes. Our study has shown that different rare histotypes impact survival, with some variants, namely MMC and DDC, showing significantly better survival than others (IMC, DAC, and mixed). This confirms previous findings relative to similar histotypes in other sites (e.g., stomach, breast, and pancreas) [30,42,43] and underscores the importance of their recognition and specific diagnosis in the pathology reports.

SRCs were found in different subtypes and did not constitute a “per se” SRC subtype in our series. ROC analysis identified a 5% cut-off value of SRCs as prognostically informative only in the univariate analysis, while this was lost in the multivariate analysis. This result partly contrasts numerous studies that have shown SRCs to present a negative prognostic impact with worse survival and response to chemotherapy [44]. A possible explanation for this discrepancy is the fact that most SRCs are found in the higher risk group, but this is not exclusive, as they may also be found in the diffuse desmoplastic carcinoma, which is part of the lower risk group. It is likely that the favorable prognostic impact of diffuse desmoplastic carcinomas is lost as the overwhelming majority of cancers with SRC morphology are high grade. Furthermore, literature is rife with methodological pitfalls for the evaluation of the impact of SRCs. Indeed, this variability is linked to: (i) the inclusion of only EA/EGJAs or these together with gastric carcinomas or cases undergoing different treatments (surgery versus neoadjuvant therapy followed by surgery), (ii) the criterion used for defining SRC, and (iii) the appropriate percentage cut off required to diagnose SRC carcinoma [39,45,46,47,48]. This topic has been extensively addressed in a recent review [44] which calculated a 7.61% rate of SRC carcinomas among 18,989 EA/EGJAs, but with a wide range, between 0.46 and 26%, in different case series.

Vascular invasion has proved to be of prognostic impact in univariate and multivariate analyses, suggesting that it is a core feature that must be reported. Of note, this parameter was identified only in routine hematoxylin and eosin sections, with no added histochemical stains or immunostaining.

Tumor budding (defined as a single cell or a non-glandular cluster of less than five cancer cells) is becoming an important prognostic parameter in different types of cancer, including EAs [49]. Budding has not, as yet, been assessed in our analysis; evaluation should (probably) only be applied to glandular carcinomas with particular distinction between true buds and non-gland-forming solid clusters of more than five cells, typical of the GL-PD subtype.

With regards to the possible interaction between adjuvant therapy and survival according to histotype and stage, no effect emerged, suggesting that adjuvant therapy was not a confounding factor.

A strength of our study is that cases were subjected to central pathology review, which guaranteed relatively uniform tumor classification. Though retrospective in nature, all cases were collected from centers with high volumes of esophageal surgery and assessed by expert gastrointestinal pathologists. None of the cases were subject to neoadjuvant treatment, making this classification applicable in the baseline setting and free of potential treatment-induced morphologic modifications.

## 5. Conclusions

In conclusion, the major novelty of this study has been the introduction of a diagnostic algorithm that separates adenocarcinomas with glandular architecture from rare histotypes, and further grades the former and subtypes the latter. This morphologic distinction has proved to have a statistically significant prognostic impact on its own or dichotomized into lower and higher risk carcinomas, especially when coupled with stage. Indeed, the stage plus histotype combination shows a high discriminating power for 5-year CSS, ranging from 87.6% in stage II lower risk group to 14% in stage IVA higher risk group. To our knowledge, none of the previous approaches have shown such a high degree of prognostic prediction as the one shown in Scheme 1. We recognize that a purely morphologic approach to EA/EGJAs may seem limited and “old fashioned” in the age of molecular pathology, however the results of a thorough and detailed analytical evaluation of morphologic subtypes has proven to be extremely encouraging for prognostic prediction. To validate results and assess ease of application, the results of this study will need to be confirmed on different series of treatment-naïve cancers with similar characteristics. Finally, as neoadjuvant therapy is becoming the standard of treatment for such tumors, the natural evolution of this study involves the evaluation of the applicability of these morphologic criteria on pre-treatment endoscopic biopsies and on surgical samples following neo-adjuvant therapy.

## Figures and Tables

**Table 1:** Main clinical and pathological characteristics of the case series.

Main Clinical Characteristics			
**Gender**	**Male**		243 (81.3%)
	**Female**		56 (18.7%)
**Age (yrs)**	**Median**		**IQR**
	72		62–78
**Siewert**	**1**	**2**	**3**
	119 (39.8%)	124 (41.5%)	56 (18.7%)
**R status**	**0**		**1**
	280 (93.6%)		19 (6.4%)
**Lymph node Number**	**Median**		**IQR**
	28		20–41
**Main Pathologic Characteristics**			
**T**	**1–2**	**3**	**4**
	95 (31.8%)	179 (59.9%)	25 (8.4%)
**Stage (pTNM)**	**Ib-II**	**III**	**IV**
	80 (26.8%)	149 (49.8%)	70 (23.4%)
**Laurén**	**Intestinal**	**Diffuse**	**Unclassified**
	250 (83.6%)	29 (9.7%)	20 (6.7%)
**Ming**	Expanding		84 (28.1%)
	Infiltrative		215 (71.9%)
**Histological subtype**	**Lower risk**		**116 (38.8%)**
	Glandular Well Differentiated		103 (34.4%)
	Mucinous Muconodular Carcinoma		8 (2.7%)
	Diffuse Desmoplastic Carcinoma		5 (1.7%)
	**Higher risk**		**183 (61.2%)**
	Glandular Poorly Differentiated		137 (45.8%)
	Invasive Mucinous Carcinoma		17 (5.7%)
	Diffuse Anaplastic Carcinoma		9 (3%)
	Mixed Carcinoma		20 (6.7%)

yrs—years; IQR—interquartile range.

**Table 2:** Main results of univariate and multivariate statistical analysis of survival in alandular adenocarcinoma.

UNIVARIATE ANALYSIS			Kaplan–Meier Analysis			Cox Regression Analysis		
**Variables**	**Criterion**	**No.** **Patients**	**CSS**		***p***	**HR**	**95% CI**	***p***
			**Censored**	**No. of Events**				
Cut off of loss of glandular structure (i.e., poorly differentiated component)	<6	103	76	27	<0.0001	2.361	1.504–3.707	<0.0001
	≥6	137	73	64				
Ming’s growth pattern	Infiltrative	165	98	67	0.011	0.551	0.345–0.881	0.013
	Expansive	75	51	24				
Vascular invasion	No	88	66	22	0.001	2.184	1.351–3.531	0.001
	Yes	152	83	69				
Perineural invasion	No	131	92	39	<0.0001	2.110	1.385–3.214	0.001
	Yes	109	57	52				
**MULTIVARIATE ANALYSIS**								
**Variables**		***p***						
Ming		0.901						
					**HR**		**95% CI**	
6% cut-off of loss of glandular structure (i.e., poorly differentiated component		0.010			1.860		1.160–2.981	
Vascular invasion		0.036			1.702		1.036–2.797	
Perineural invasion		0.040			1.588		1.020–2.472	

CSS—cancer specific survival; HR—hazard ratio; CI—confidence interval.

**Table 3:** Results of univariate and multivariate statistical analysis of survival.

UNIVARIATE ANALYSIS								
			Kaplan–Meier Analysis			Cox regression Analysis		
**Variables**	**Criterion**	**No.** **Patients**	**CSS**		***p***	**HR**	**95% CI**	***p***
			**Censored**	**No. of Events**				
Age (yrs)	<72	148	93	55	0.013	1.580	1.095–2.279	0.015
	≥72	151	90	61				
Signet ring	<5%	246	156	90	0.030	1.610	1.039–2.495	0.033
	≥5%	53	27	26				
Non Cohesive cells	No	221	142	79	0.014	1.617	1.094–2.392	0.016
	Yes	78	41	37				
Ming’s growth pattern	Infiltrating	215	125	90	0.004	0.534	0.344–0.827	0.005
	Expanding	84	58	26				
Lauren’s classification	Intestinal	250	157	93	0.089	1.479	0.935–2.338	0.094
	Diffuse/ Unclassified	49	26	23				
Vascular Invasion	No	107	80	27	<0.0001	2.242	1.456–3.450	<0.0001
	Yes	192	103	89				
Perineural Invasion	No	165	112	53	0.001	1.883	1.303–2.721	0.001
	Yes	134	71	63				
Stage (pTNM)	I/II	80	63	17	<0.0001	2.337	1.785–3.060	<0.0001
	III	149	89	60				
	IV	70	31	39				
Histologic classification	Lower risk	116	86	30	<0.0001	2.415	1.593–3.664	<0.0001
	Higher risk	183	97	86				
**MULTIVARIATE ANALYSIS**								
**Variables**		***p***						
Signet ring		0.297						
Non-cohesive cells		0.250						
Ming’s growth pattern		0.824						
Lauren’s classification		0.671						
Perineural Invasion		0.245						
					**HR**		**95% CI**	
Stage (pTNM)		<0.0001			2.089		1.588–2.749	
Histologic classification		0.001			2.047		1.336–3.135	
Age		0.008			1.659		1.142–2.411	
Vascular Invasion		0.015			1.726		1.113–2.677	

yrs—years; CSS—cancer-specific survival; HR—hazard ratio; CI—confidence interval.

## Data Availability

The datasets used and analyzed during the current study are available from the corresponding author on reasonable request.
